# Mental and physical health characteristics of older and younger adults receiving medication for opioid use disorder

**DOI:** 10.3389/fpubh.2024.1418690

**Published:** 2024-11-12

**Authors:** Teresa J. Walker, Rakshitha Mohankumar, Shane W. Kraus, Brandi P. Cotton, Brenna N. Renn

**Affiliations:** ^1^Department of Psychology, University of Nevada, Las Vegas, NV, United States; ^2^Department of Psychiatry, Yale School of Medicine, New Haven, CT, United States

**Keywords:** opioids, older adults, depression, anxiety, self-rated health, medication for opioid use disorder, methadone

## Abstract

**Background:**

Methadone is an effective and widely used medication for opioid use disorder (MOUD). Within in the United States (US), older adults represent an increasing proportion of those receiving MOUD, yet little is known about characteristics of older individuals in these programs.

**Objectives:**

To evaluate mental and physical health characteristics of younger and older adults receiving MOUD and test whether age moderates the relation between physical and mental health variables.

**Methods:**

Data for this secondary analysis are drawn from a cross-sectional survey of a convenience sample of individuals seeking methadone dosing as part of MOUD at four opioid treatment programs in two regions of the US. Descriptive statistics and correlational and moderation analyses examined outcomes of pain severity, pain interference, self-rated health, physical activity, depression, and anxiety across younger (18-49) and older (50+ years) participants.

**Results:**

Analyses included 469 participants (mean [range] age, 41.01 [20–70] years). Older participants reported higher pain severity and interference, less physical activity, and worse self-rated health than those in the younger age group (*p*s < 0.05). Associations between mental and physical health variables were generally weak-to-moderate in the older age group (*r* = 0.26 to *r* = 0.44, *p* < 0.01), and weak in the younger age group, although age did not moderate associations.

**Conclusion:**

Clinically significant mental and physical health variables are associated among individuals receiving methadone for MOUD, with older adults facing unique challenges.

**Clinical implications:**

Opioid use treatment should include not only pain management but also assessment and treatment of depression and anxiety and optimization of other health behaviors (e.g., physical activity) across age groups. Pain management and health promotion are particularly relevant targets for aging individuals receiving MOUD.

## 1 Introduction

The opioid epidemic in the United States (US) is steadily increasing with opioid-involved overdose deaths escalating from 47,000 in 2018 to almost 70,000 in 2020 (1). Methadone is an effective and widely used medication for opioid use disorder (MOUD) typically offered through opioid treatment programs (OTPs) (2). As in many facets of healthcare, there is a corresponding surge in the number of older adults receiving OUD (3–5). This demographic shift is driven in part by cohort effects such as increased heroin availability and use in the 1960s and 1970s and the beginning of the opioid epidemic in the 1990s (6, 7) coupled with improved access to healthcare and treatment services, which has afforded longer lifespans (8). Treatment can be initiated at any age and some individuals may require long-term—even lifelong—maintenance. Thus, older adults receiving MOUD reflect both individuals who have aged with OUD and those newly acquiring OUD in middle or later life. Despite elevated OUD among adults 50 and older, medical and mental health comorbidities and treatment considerations among older adults with OUD have historically received little attention (5, 9–12).

Both older and younger individuals utilizing methadone in MOUD programs tend to have high levels of disease burden and poor general health compared to population norms (13–15). Depressive and anxiety symptoms are especially common among younger and middle aged individuals (16, 17) and older adults (15, 18) with substance use disorders (SUDs), including OUD and those utilizing methadone in MOUD, relative to average population prevalence. These co-occurring conditions are associated with lower initiation and retention in treatment and an increased risk for relapse across adult populations (19). However, evidence is limited and inconclusive regarding age differences in health status among individuals using MOUD. While older adults in the general US population often have better mental health than their younger counterparts, including lower prevalence of diagnosable (“clinical”) mental health conditions (20), some examinations of individuals prescribed methadone in MOUD showed no differences between older and younger adults in terms of endorsement of psychiatric symptoms (14, 21). Even subsyndromal depressive symptoms in older adults can impart functional decline, greater comorbidity, frailty, cognitive decline, and greater demand for healthcare services (22–24). Mental health problems among older adults are often and disproportionally associated with physical health concerns (25) and this relationship becomes more complex as individuals age and face increasing health concerns (26). Age is an important predictor of a poorer course of depressive (27) and anxiety (28) disorders for other reasons, including loneliness and loss of social support in addition to associated health vulnerabilities such as pain, cognitive impairment, and comorbid chronic disease and related inflammation (27). These consistent findings illustrate that the biological underpinnings linking physical, cognitive, and mental health are pronounced in aging and may suggest a stronger relationship between mental and physical health with age.

Despite the US demography shifts and evidence that older adults are more likely to need and seek treatment for substance use disorders, including OUD, much of the substance use literature focuses on alcohol use and excludes older adult participants (29). The field has largely failed to examine older adults with OUD and those receiving MOUD. Several age-related factors, including co-occurring chronic disease and declining health, chronic pain, functional ability, social isolation, and stigma may influence an individual’s success or satisfaction with methadone in MOUD (30). Older adults are more likely to report greater pain severity (31), and pain during OUD treatment is associated with poorer treatment outcomes like relapse and lower retention in OTPs (32). Conversely, older age is associated with a greater likelihood of retention in OTPs (33), which is a leading predictor of positive treatment outcomes, including reduction of relapse and high-risk behaviors (34). Until very recently, the existing literature on older adults in OTPs receiving methadone was generally limited to single site data collection (e.g., from one clinic in a US city) and characterized by small sample size (e.g., 24–54 participants) (12). Work by Rosen and colleagues (15, 35) included larger samples (i.e., 140 individuals aged 50 and older), albeit from one US city. Although preliminary, this body of work has documented a high prevalence of mental health disorders (e.g., major depressive disorder, generalized anxiety disorder), prevalent chronic physical disease (e.g., arthritis, hypertension), and subjective self-perception of having fair to poor physical health among the majority of the older adult respondents. More recently, a pilot study comparing older adults receiving methadone to a national population-based cohort revealed significantly higher prevalence of conditions including visual and hearing impairment, incontinence, chronic pain, and falls than the national population-based cohort (13).

Aging individuals who have used heroin, other opiates, and opioids experience an accelerated aging process and have complex accumulated health needs. Additionally, long-term opioid use for chronic pain can develop into OUD (36), and chronic pain is often experienced in older adults and individuals on MOUD. However, comparisons between age groups and a lifespan perspective on these clinical profiles is lacking. A better understanding of the unique profiles of older vs. younger adults utilizing methadone in MOUD may help researchers and practitioners develop strategies to engage and effectively treat these individuals.

The current study had two primary aims: (1) to describe mental and physical health characteristics of younger and older adults utilizing methadone in MOUD and test the hypothesis that older adults will report worse mental and physical health characteristics than younger adults; and (2) to conduct an exploratory test of whether age moderated relationships between physical and mental health variables among adults utilizing methadone in MOUD. Although we expect mental and physical health to be positively related in both younger and older groups, we propose to test a moderation model to identify whether the strength of this relationship varies by age group. The dearth of literature on aging adults in MOUD—and among substance use in older adults generally—calls for more examination into potential effect modifiers to better understand risk and resilience and inform targeted needs for assessment and intervention.

## 2 Method

### 2.1 Design, participants, and procedures

Data were drawn for secondary analysis from a cross-sectional study of adults enrolled in OTPs, conducted in two US regions in 2017. Reporting follows the STROBE statement guidelines (37). The original study enrolled 484 participants who were recruited from four OTPs in Southern New England (*n* = 384) and the Pacific Northwest (*n* = 100). Using convenience sampling, clinic patients were invited by the investigators and clinical staff to complete a self-administered paper survey assessing sociodemographic and health information, substance use, psychosocial functioning, and MOUD characteristics and outcomes. Individuals were excluded from participation if they were younger than 18 years old (and therefore were not able to receive methadone) or were not fluent in English. Participants provided informed consent and the study was approved by the Institutional Review Boards of Dartmouth College and Spectrum Health Services.

To answer the research questions of this secondary analysis, we removed 16 individuals with no age data from the original sample. Compared to the included sample (*N* = 469), the 16 excluded individuals had greater pain interference (*M* = 3.95 vs. 5.75, *p* = 0.048) and lower levels of past 6-month physical activity (*M* = 2.36 vs. 3.50, *p* = 0.010). Those included vs removed from analysis on the basis of missing age data did not differ on other outcomes of interest.

### 2.2 Measures

#### 2.2.1 Participant characteristics and sample classification

##### 2.2.1.1 Sociodemographic characteristics

Participants self-reported basic demographic information including age (in years), gender (*man/male, woman/female*, or *gender nonconforming*), race (*American Indian/Alaskan Native*, *Black or African American*, *Native Hawaiian or other Pacific Islander*, *Asian*, *White*, or *multiracial*), ethnicity (*Hispanic/Latino* or *Non-Hispanic/Latino*), and employment status (*Unemployed*, *part-time*, *full-time*, or *on disability*).

##### 2.2.1.2 Treatment variables

Participants self-reported the option which best described why they began an OTP (*I was FIRST addicted to painkillers and then became addicted to heroin*; *I was FIRST addicted to heroin and then became addicted to painkillers*; *I was addicted to painkillers but never used heroin*; *I was addicted to heroin but never abused painkillers*; *I was NEVER addicted, I take methadone ONLY for pain*).

#### 2.2.2 Health-related variables

##### 2.2.2.1 Pain

Two items assessing pain intensity/severity (0 = *no pain* to 10 = *most intense*) and pain interference (0 = *none* to 10 = *extreme interference*) were drawn from the West Haven-Yale Multidimensional Pain Inventory (38). This measure has demonstrated reliability and convergent validity (39).

##### 2.2.2.2 Self-rated health

A single item (“In general, would you say your health is…) measured self-rated health from 1 (*excellent*) to 5 (*poor*). This measure is valid, reliable, and widely used in epidemiological and survey research (40).

##### 2.2.2.3 Physical activity

Self-reported physical activity was measured using the 6-level physical activity scale, “How physically active do you think you have been during the last six months?” (41). Physical activity was classified by one of the following responses, 1 = *hardly any physical activity* through 6 = *hard or very hard exercise regularly and several times a week*. Responses between 4–6 correspond to the World Health Organization’s recommended levels of physical activity (42). This scale has demonstrated good psychometric characteristics, including among older adults and others with lower levels of physical activity (43).

##### 2.2.2.4 Mental health

The presence and frequency of depression and anxiety symptoms were assessed using the 4-item Patient Health Questionnaire (PHQ-4) (44). The PHQ-4 is a self-report instrument consisting of two core depression items from the PHQ-9 (45) and two core anxiety items from the Generalized Anxiety Disorder (GAD-7) scale (46). Responses to each item ranged from 0 (*not at all*) to 3 (*nearly every day*). The PHQ-4 is commonly used in survey research to briefly screen for the presence of depressive and anxiety symptoms and has demonstrated reputable internal reliability, construct validity, and discriminant validity (44). Separate depression and anxiety symptom scales were calculated by summing the two depression or anxiety items, respectively. Additionally, total depression and anxiety symptoms (hereinafter referred to as *mental health*) were determined for each participant by adding together the scores of all 4 items. Higher scores indicate higher levels of depressive symptoms, anxiety symptoms, and mental health symptoms.

### 2.3 Statistical analysis

Given the accelerated aging and premature mortality associated with substance use, there is no current consensus regarding the cutoff for “older” adults in the substance use literature (8, 47). We compared participants between ages 18 and 49 years (“younger adults”) to participants ages 50 years and older (“older adults”) based on prior studies of SUDs in older adulthood that generally include adults ages 50 years and older (48, 49). Given the difference in sample sizes between younger and older adults, we used Levene’s test to assess homogeneity of variance in all outcome variables between the two age groups. All outcome variables met the threshold for equal variances assumed (*p* > 0.05) with the exception of anxiety symptoms as measured by the GAD-2 (*p* = 0.04). GAD-2 scores were thus subjected to square root transformation to equalize or stabilize variance; once transformed, Levene’s test supported equality of variance (*p* = 0.06). All statistical tests described below were analyzed using both the raw GAD-2 continuous score and the transformed GAD-2 score; results did not change. For the purpose of parsimony and ease of interpretation, the results of the untransformed (raw) GAD-2 measure are reported throughout.

Prior to all analyses, the data were reviewed for other general assumptions of parametric tests (50). Normality was assessed by skewness and kurtosis values between −2 and + 2 and aided by visual inspection of univariate normal Q-Q plots and histograms. For all regression analyses (moderation analyses, see below), normality was further assessed via inspection of normal P–P plot of regression standardized residuals and linearity assessed via scatterplot of standardized residuals. Data on all outcomes were sufficiently linear. Outliers were infrequent and such cases were retained as analysis with and without these values did not affect the results. Summary statistics described the sample. Correlations between the primary variables of interest (i.e., age group, pain, mental health variables, self-rated health, and physical activity) were calculated using Pearson’s correlation for continuous variables, Spearman’s correlations for ordinal variables, and Pearson’s chi-square for categorical variables. Strength of correlations were interpreted according to existing guidelines (51) as 0.0 to 0.2 = *very weak*, 0.2 to 0.4 = *weak*, 0.4 to 0.6 = *moderate*, 0.6 to 0.8 = *strong* (absolute values). 95% confidence intervals (CI) were added to all correlations in brackets.

Moderation analyses tested the effect of age on the associations of physical health and pain variables with depression symptoms, anxiety symptoms, and mental health. Specifically, the moderating effect of age was tested for the following associations: (1) self-rated health and depression, (2) self-rated health and anxiety, (3) self-rated health and aggregate mental health, (4) physical activity and depression, (5) physical activity and anxiety, (6) physical activity and mental health, (7) pain severity and depression, (8) pain severity and anxiety, (9) pain severity and mental health, (10) pain interference and depression, (11) pain interference and anxiety, and (12) pain interference and mental health. Age was kept as a continuous variable for moderation analyses to increase sensitivity for detecting a significant effect. All statistical analyses were performed using IBM SPSS Statistics version 28.0 and version 4.0 of the PROCESS macro for SPSS (52).

G power analyses for linear regression were performed *post hoc* to calculate observed power to detect a moderation effect of age in our sample (53). Observed statistical power across models was >0.95.

## 3 Results

### 3.1 Participant characteristics

Participant characteristics are presented in Table 1. The mean age of the sample was 41 years (*SD* = 11.61), and 74.60% of the sample were less than 50 years old (*n* = 350). Over half of the sample identified as men (55.60%, *n* = 266) and White (87.60%, *n* = 383). Compared to those younger than 50 years old, participants in the older age group were more likely to be on disability (15.6% vs. 51.7%, *p* < 0.001) and less likely to have entered initial treatment with a painkiller addiction before acquiring a heroin addiction (67.9% vs. 46.2%, *p* < 0.001). Older adults also reported higher pain severity (*M* = 4.32 vs. *M* = 5.36, *p* = 0.001), higher pain interference (*M* = 3.51 vs. *M* = 5.35, *p* < 0.001), worse self-rated health (*M* = 3.24 vs. *M* = 3.60, *p* < 0.001), and less physical activity in the past 6 months (*M* = 3.59 vs. *M* = 3.18, *p* = 0.019), compared to their younger counterparts in the sample.

**Table 1:** Participant characteristics.

Variable	Total sample		Ages 18–49 years		Ages ≥ 50 years		
	(*n* = 469)		(*n* = 350)		(*n* = 119)		
	Frequency	Range	Frequency	Range	Frequency	Range	*p*-value
Age (*N* = 469), mean, (SD)	41.04 (11.6)	20–70	35.52 (7.1)	20–49	57.29 (5.38)	50–70	
Age group, no. (%)							
Less than 50 years	350 (74.6)						
50 years and older	119 (25.4)						
Gender (*N* = 462), no. (%)							
Man/male	256 (55.4)		187 (54.2)		69 (59.0)		
Woman/female	204 (44.2)		156 (45.2)		48 (41.0)		
Gender nonconforming	2 (0.4)		2 (0.6)		0 (0.0)		
Race (*N* = 425), no. (%)							
American Indian/Alaskan native	13 (3.1)		5 (1.6)		8 (7.6)		
Black or African American	14 (3.3)		4 (1.3)		10 (9.5)		
Native Hawaiian or other pacific Islander	5 (1.2)		5 (1.6)		0 (0.0)		
Asian	1 (0.2)		1 (0.3)		0 (0.0)		
White	374 (88.0)		291 (90.9)		83 (79.0)		
Multiracial	18 (4.2)		14 (4.4)		4 (3.8)		
Ethnicity (*N* = 377), no. (%)							
Hispanic/Latino	51 (13.5)		37 (12.9)		14 (15.4)		
Non-Hispanic/Latino	326 (86.5)		249 (87.1)		77 (84.6)		
Employment status (*N* = 462), no. (%)							
Unemployed	165 (35.7)		132 (38.2)		33 (28.4)		
Part-time	77 (16.7)		62 (17.9)		15 (12.9)		
Full-time	106 (22.9)		98 (28.3)		8 (6.9)		
On disability	114 (24.7)		54 (15.6)		60 (51.7)		
Reason for entering treatment (*N* = 452), no. (%)							
Painkillers then heroin	284 (62.8)		235 (67.9)		49 (46.2)		
Heroin then painkillers	32 (7.1)		17 (4.9)		15 (14.2)		
Heroin only	85 (18.8)		59 (17.1)		26 (24.5)		
Painkillers only	43 (9.5)		35 (10.1)		8 (7.5)		
Pain only (no SUD)	8 (1.8)		0 (0.0)		8 (7.5)		
Age at first use (*N* = 422), mean, (SD)	22.91 (8.7)	8–60	21.27 (6.4)	10–47	28.51 (12.4)	8–60	
Pain severity^a^ (*N* = 443), mean, (SD)	4.56 (2.9)	0–10	4.31 (2.8)	0–10	5.36 (3.0)	0–10	0.001
Pain interference^b^ (*N* = 447), mean, (SD)	3.95 (3.1)	0–10	3.51 (2.9)	0–10	5.35 (3.2)	0–10	<0.001
Self-rated health^c^ (*N* = 442), mean, (SD)	3.33 (0.9)	1–5	3.24 (0.9)	1–5	3.60 (1.0)	1–5	<0.001
Past 6-month physical activity^d^ (*N* = 396), no. (%)							0.019
Hardly any physical activity	43 (10.9)		30 (9.7)		13 (14.9)		
Mostly sitting, sometimes a walk or light gardening	52 (13.1)		38 (12.3)		14 (16.1)		
Light physical exercise around 2–4 h/week	100 (25.3)		76 (24.6)		24 (27.6)		
Moderate exercise 1–2 h/week, or > 4 h of light activity	108 (27.3)		87 (28.2)		21 (24.1)		
Moderate exercise at least 3 h/week	50 (12.6)		40 (12.9)		10 (11.5)		
Hard or very hard exercise regularly and several times/week	43 (10.9)		38 (12.3)		5 (5.7)		
Total depression and anxiety score^e^ (*N* = 414), mean, (SD)	5.32 (3.7)	0–12	5.30 (3.8)	0–12	5.41 (3.4)	0–12	0.792
Depression score^f^ (*N* = 418), mean, (SD)	2.62 (1.9)	0–6	2.58 (2.0)	0–6	2.74 (1.9)	0–6	0.471
Anxiety score^g^ (*N* = 430), mean, (SD)	2.71 (2.0)	0–6	2.73 (2.1)	0–6	2.65 (1.9)	0–6	0.753

SUD = substance use disorder. ^a^Pain Severity was measured using the West Haven-Yale Multidimensional Pain Inventory and scores range from 0 (no pain) to 10 (most intense). ^b^Pain Interference was also measured using the West Haven-Yale Multidimensional Pain Inventory and scores range from 0 (none) to 10 (extreme interference). ^c^Self-rated Health was measured from a single item where scores ranged from 1 (excellent) to 5 (poor). ^d^Past 6-Month Physical Activity was measured using the 6-level Physical Activity Scale where scores ranged from 1 (hardly any physical activity) to 6 (hard or very hard exercise regularly and several times a week). Responses of 4–6 correspond to the World Health Organization’s recommended levels of activity. ^e^Total Depression and Anxiety score was measured using the four-item Patient Health Questionnaire (PHQ-4) for anxiety and depression. Total score is determined by adding together the scores of each of the 4 items. Scores are rated as normal (0–2), mild (3-5), moderate (6-8), and severe (9-12). ^f,g^Depression and Anxiety scores were also separately calculated based on individual items from the PHQ-4; total scores of ≥ 3 for either suggest depression or anxiety, respectively.

The overall sample reported mild-to-moderate symptoms of depression and anxiety on the PHQ-4 (*M* = 5.34, *SD* = 3.7). Endorsement of depressive symptoms (younger: *M* = 2.58 [*SD* = 2.0] vs. older: *M* = 2.74 [*SD* = 1.9], *p* = 0.471) and anxiety symptoms (younger: *M* = 2.73 [*SD* = 2.1] vs. older: *M* = 2.65 [*SD* = 1.9], *p* = 0.75) was not significantly different across age groups.

### 3.2 Associations between health-related variables

Main correlations are presented in Table 2. Collapsing across the entire sample, bivariate correlations revealed a weak-to-moderate association between worse self-rated health and greater depression (*r* = 0.33 [0.27, 0.44]), greater anxiety (*r* = 0.27 [0.19, 0.37]), higher pain severity (*r* = 0.29 [0.22, 0.40]), and higher pain interference (*r* = 0.37 [0.29, 0.45]); all *p*s < 0.001. Similarly, less physical activity was weakly associated with greater depression (*r* = −0.26 [−0.35, −0.16]), greater anxiety (*r* = −0.14 [−0.23, − 0.04]), higher pain severity (*r* = −0.16 [−0.27, −0.08]), and higher pain interference (*r* = −0.21 [−0.31, −0.12]) all *p*s < 0.01. Correlation analyses also showed that higher pain severity and higher pain interference were weak-to-moderately associated with greater depression (*r* = 0.18 [0.08, 0.27] and *r* = 0.21 [0.12, 0.30], both *p*s < 0.001) and greater anxiety (*r* = 0.24 [0.14, 0.32] and *r* = 0.24 [0.15, 0.33], both *p*s < 0.001). Finally, greater mental health symptomatology demonstrated positive weak associations with pain severity (*r* = 0.22 [0.12, 0.31]) and pain interference (*r* = 0.24 [0.15, 0.33], both *p*s < 0.001).

**Table 2:** Bivariate correlations.

	Age group	Total depression and anxiety score	Depression score	Anxiety score	Self-rated health	Past 6-month physical activity	Pain severity	Pain interference
**Age group	1.00	0.01	0.04	−0.02	**0.16**	**−0.12**	**0.16**	**0.26**
Total depression and anxiety score	–	1.00	**0.92**	**0.93**	**0.33**	**−0.22**	**0.22**	**0.24**
Depression score	–	–	1.00	**0.70**	**0.33**	**−0.26**	**0.18**	**0.21**
Anxiety score	–	–	–	1.00	**0.27**	**−0.14**	**0.24**	**0.24**
*Self-rated health	–	–	–	–	1.00	**−0.31**	**0.29**	**0.35**
*Past 6-month physical activity	–	–	–	–	–	1.00	**−0.17**	**−0.22**
Pain severity	–	–	–	–	–	–	1.00	**0.80**
Pain interference	–	–	–	–	–	–	–	1.00

Bold = *p* significant at 0.01 level. * Two-tailed Spearman’s rho correlation. ** Two-tailed point biserial Pearson correlation.

Among older adults, worse mental health was associated with greater pain severity (*r* = 0.26 [0.05, 0.45], *p* = 0.016) and pain interference (*r* = 0.42 [0.23, 0.58], *p* < 0.001), worse self-rated health (*r* = 0.44 [0.26, 0.59], *p* < 0.001), and less physical activity (*r* = −0.26 [−0.45, −0.04], *p* = 0.023). These associations were weak for younger adults in the sample (mental health and pain severity: *r* = 0.20 [0.10, 0.31], *p* < 0.001; pain interference: *r* = 0.20 [0.09, 0.30], *p* < 0.001; self-rated health *r* = 0.33 [0.23, 0.43], *p* < 0.001; physical activity *r* = − 0.21 [−0.31, −0.10], *p* < 0.001). For the older adults in this sample, worse self-rated health was associated with less past 6-month physical activity (*r* = −0.26 [−0.45, −0.05], *p* = 0.016), greater pain severity (*r* = 0.33 [0.14, 0.50], *p* < 0.001), and greater pain interference (*r* = 0.42 [0.25, 0.57], *p* < 0.001). Younger adults also evidenced a weak relationship between worse self-rated health and less past 6-month physical activity (*r* = −0.31 [−0.41, −0.21], *p* < 0.001), greater pain severity (*r* = 0.28 [0.18, 0.38], *p* < 0.001), and pain interference (*r* = 0.32 [0.22, 0.41], *p* < 0.001).

### 3.3 Moderating effect of age

Moderation analyses were performed to assess the indirect effect of age on the relationships between self-rated health, past 6-month physical activity, pain severity, pain interference, and depression symptoms, anxiety symptoms, and mental health. Age did not moderate any of these relations between variables (all *p*-values >0.10; Table 3).

**Table 3:** Results of regression models testing moderating effect of age on physical health and mental health variables.

	Coeff	SE	*t*	*p*	95% CI	Δ*R^2^*
Model 1 (outcome = depression score [PHQ2])						
Self-rated health	0.73	0.10	7.34	0.000	[0.53, 0.92]	
Age	0.00	0.01	0.48	0.631	[−0.01, 0.02]	
Self-rated health x age	0.00	0.01	0.09	0.929	[−0.02, 0.02]	0.0000
Model 2 (outcome = depression score [PHQ2])						
Past 6-month physical activity	−0.33	0.07	−4.87	0.000	[−0.46, −0.20]	
Age	0.02	0.01	2.12	0.035	[0.00, 0.04]	
Past 6-month physical activity x age	0.00	0.01	0.27	0.787	[−0.01, 0.01]	0.0002
Model 3 (outcome = depression score [PHQ2])						
Pain severity	0.11	0.04	3.12	0.002	[0.04, 0.18]	
Age	0.01	0.01	1.01	0.315	[−0.01, 0.03]	
Pain severity x age	−0.00	0.00	−0.22	0.824	[−0.01, 0.01]	0.0001
Model 4 (outcome = depression score [PHQ2])						
Pain interference	0.13	0.03	3.71	0.000	[0.06, 0.19]	
Age	0.00	0.01	0.39	0.697	[−0.01, 0.02]	
Pain interference x age	0.00	0.00	0.77	0.444	[−0.00, 0.01]	0.0014
Model 5 (outcome = anxiety score [GAD2])						
Self-rated health	0.62	0.10	5.96	0.000	[0.41, 0.82]	
Age	0.00	0.01	0.10	0.924	[−0.02, 0.02]	
Self-rated health x age	−0.00	0.01	−0.38	0.705	[−0.02, 0.01]	0.0003
Model 6 (outcome = anxiety score [GAD2])						
Past 6-month physical Activity	−0.19	0.07	−2.58	0.010	[−0.33, −0.04]	
Age	0.01	0.01	1.08	0.279	[−0.01, 0.03]	
Past 6-month physical activity x age	0.00	0.01	0.23	0.816	[−0.01, 0.01]	0.0001
Model 7 (outcome = anxiety score [GAD2])						
Pain severity	0.16	0.04	4.42	0.000	[0.09, 0.23]	
Age	0.00	0.01	0.32	0.746	[−0.01, 0.02]	
Pain severity x age	0.00	0.00	0.57	0.570	[−0.00, 0.01]	0.0007
Model 8 (outcome = anxiety score [GAD2])						
Pain interference	0.17	0.03	4.82	0.000	[0.10, 0.23]	
Age	−0.00	0.01	−0.49	0.627	[−0.02, 0.01]	
Pain interference x age	0.00	0.00	0.48	0.635	[−0.00, 0.01]	0.0005
Model 9 (outcome = total depression and anxiety score [PHQ-4])						
Self-rated health	1.37	0.19	7.32	0.000	[1.00, 1.74]	
Age	0.01	0.02	0.39	0.698	[−0.02, 0.04]	
Self-rated health x age	−0.00	0.02	−0.04	0.971	[−0.03, 0.03]	0.0000
Model 10 (outcome = total depression and anxiety score [PHQ-4])						
Past 6-month physical activity	−0.53	0.13	−4.08	0.000	[−0.79, −0.28]	
Age	0.03	0.02	1.83	0.067	[−0.00, 0.06]	
Past 6-month physical activity x age	0.00	0.01	0.15	0.882	[−0.02, 0.03]	0.0001
Model 11 (outcome = total depression and anxiety score [PHQ-4])						
Pain severity	0.26	0.07	3.93	0.000	[0.13, 0.39]	
Age	0.01	0.02	0.73	0.463	[−0.02, 0.05]	
Pain severity x age	0.00	0.01	0.25	0.806	[−0.01, 0.01]	0.0001
Model 12 (outcome = total depression and anxiety score [PHQ-4])						
Pain interference	0.28	0.06	4.39	0.000	[0.15, 0.40]	
Age	0.00	0.02	0.10	0.922	[−0.03, 0.04]	
Pain interference x age	0.00	0.01	0.67	0.502	[−0.01, 0.01]	0.0011

## 4 Discussion

The present study assessed the distinct profile of older adults in an OTP by describing mental and physical health characteristics across age groups and testing whether older adults differed from their younger counterparts in our sample on health-related variables. We also tested whether age moderated the relationships between these characteristics. Older adults in our sample reported significantly worse overall physical health, pain severity, and pain interference compared to younger adults. Across the sample, worse self-rated health and less physical activity were associated with greater depression and anxiety and higher pain severity and interference. Higher pain severity and interference were also significantly associated with greater depression and anxiety symptoms. Finally, the relationships between worse mental health and worse self-rated health, less physical activity, greater pain severity, and greater pain interference were generally of weak-to-moderate strength in the older adult group and weak among the younger adult group. However, age did not moderate the associations between variables.

We found support for the hypothesis that older adults receiving MOUD (that is, those who are aging with long-term use of heroin and other narcotics) would be in worse physical health than younger adults receiving MOUD. The sample on average endorsed mild-to-moderate symptoms of depression and anxiety; however, differences in mental health between younger and older participants in our sample were not significant. This extends prior works on adults with substance use disorders, which have primarily focused on alcohol use, to address the unique facets of older adults with OUD maintained on methadone (29, 54). Findings from the current study align with prior work demonstrating that older adults with OUD had poorer physical health compared to younger adults (8, 14, 55). We also found that older adults had greater pain severity and interference compared to the younger group, which may be encapsulated in poorer self-rated health ratings among older adults. These findings are important given that the presence of pain (32) and mental health symptoms (19) have been associated with lower substance use treatment retention, while older age has been associated with higher treatment retention (33). Future work should examine whether older age may mitigate the relationships between physical and mental health and treatment retention.

Despite the lack of statistical significance in the moderation analyses, it is possible—and worthy of future research—that the relationship between mental and physical health characteristics may be stronger among older vs. younger adults receiving MOUD. One possible explanation for this finding is that perceptions of health may vary by age, such that older adults may attribute mental health concerns more strongly to physical health problems compared to younger adults. Mental health presentations in older adults may be subtle or nonspecific and thus overlooked (56). Mental and physical health difficulties also vary in presentation, complexity, and experience across the life course, with older adults more likely to have medical and psychiatric comorbidity and overlapping symptom presentation. The physical health burden of mental health symptoms is magnified across the lifespan, with vulnerabilities of frailty and functional decline more likely among older than younger adults. An example of this in our sample was the moderate relation between worse mental health and pain interference (a self-report assessment of functioning) in older adults compared to the weak correlation found in the younger sample. The allostatic load framework refers to the accumulation of physiological disturbances (e.g., activation of the hypothalamic–pituitary–adrenal [HPA] axis) in response to stress and the long-term effects of such dysregulation on mental and physical health (57). This presents one possible pathway though which mental and physical health are linked, perhaps more strongly in older adults with greater allostatic load. The presence of co-occurring SUD further complicates this clinical picture; however, the intersection of age and SUD is severely understudied. An older review of 20 leading journals in substance and abuse and gerontology (10 each) found a paucity of articles with a joint focus on older adults and substance abuse (broadly considered; less than 1% of all articles) (48). Principles of geriatric medicine have yet to be incorporated into substance use treatment on a large scale, and geriatric providers are often not focused on or feel ill-equipped to treat SUDs in older adults. To adequately prepare clinicians for working with older adults who use substances, including those with OUD, further research on age-related factors in risk and treatment, as well as a coordinated effort between disciplines and practitioners, is crucial.

### 4.1 Limitations

This study provides further insights into the distinct characteristics of individuals with OUD who are maintained on methadone across age groups; however, several limitations should be carefully considered. This was a cross-sectional study, prohibiting causal associations. Future studies should consider whether these findings hold over time and across birth cohorts (e.g., among those 65 years and older and older versus 50-64-year-olds). These data were drawn from a convenience sample and may not represent the general adult population in OTPs. For example, those with more severe mental health conditions may be missing from the sample. Related, mental and physical health data are based on self-report rather than clinical data, which may have introduced bias. Future studies may consider methodology such as chart review to include a more representative sample and collect objective measures, such as diagnostic interviewing and standard physical health markers. We do not have data on survey response rates or whether respondents were representative of the OTP clinic clientele census, nor do we have data on how mental and physical health variables related to key outcomes such as treatment retention or substance use among those receiving methadone for MOUD. The majority of the sample were non-Hispanic White individuals, limiting generalizability. However, this study included a larger sample than most previous studies of older adults in OTPs, and utilized data collected from multiple cities across two distinct US regions. Despite these shortcomings, these findings contribute to the growing knowledge of the diverse characteristics and corresponding needs across individuals in treatment for OUD and lay a foundation for future research to continue to build on the profile of older adults in OTPs.

### 4.2 Summary

Significant associations between mental and physical health characteristics exist for individuals utilizing methadone for medication for OUD, although age does not appear to moderate these effects in this sample. Older adults utilizing methadone have some similarities (similar mental health symptoms) and differences (more pain, worse self-rated health) compared to their younger adult counterparts, highlighting the need to better understanding these characteristics in aging individuals in OTPs to maximize intervention effectiveness and engagement.

## Data Availability

Publicly available datasets were analyzed in this study. This data can be found here: available upon request from Brandi P. Cotton (brandi.cotton@yale.edu).
